# A rare cause of lower abdominal and pelvic mass, primary tuberculous psoas abscess: a case report

**DOI:** 10.1186/1757-1626-2-182

**Published:** 2009-11-03

**Authors:** Ana Paula Vaz, Joana Gomes, Joana Esteves, Aurora Carvalho, Raquel Duarte

**Affiliations:** 1 Department of Pulmonology, Hospital Sao Joao, Alameda Professor Hernani Monteiro 4300-319, Oporto, Portugal; 2 Department of Pulmonology, Centro Hospitalar de Vila Nova de Gaia/Espinho, Rua Conceicao Fernandes, 4434-502, Vila Nova de Gaia, Portugal; 3 Department of General Surgery, Centro Hospitalar de Vila Nova de Gaia/Espinho, Rua Conceicao Fernandes, 4434-502, Vila Nova de Gaia, Portugal; 4 Centro de Diagnostico Pneumologico de Vila Nova de Gaia, Rua Conselheiro Veloso da Cruz, 4400-092, Vila Nova de Gaia, Portugal; 5 Oporto Medical Faculty, Oporto University, Alameda Professor Hernani Monteiro 4300-319, Oporto, Portugal

## Abstract

**Introduction:**

Tuberculous psoas abscess was usually associated to complicate Pott's disease, but it can also be secondary to direct extension from other adjacent structures or haematogenous spread from an occult source. However, the occurrence of this entity as the presenting manifestation of tuberculosis, without evidence of active infection elsewhere, has been seldom reported.

**Case Presentation:**

We report a clinical case of a 64-year-old immunocompetent female that presented with left lower abdominal pain and a soft tissue mass over the left iliac fossa and inguinal regions due to a primary tuberculous psoas abscess. Early diagnosis and prompt treatment with percutaneous drainage guided by ultrasound along with antituberculous drugs, lead to a satisfactory outcome.

**Conclusion:**

The purpose of this case report is to point out attention to the diagnostic challenge of tuberculous psoas abscess in the absence of tuberculosis in other organs or a predisposing condition. A brief review of the literature about its epidemiology, etiology, clinical features and management is discussed over the text.

## Introduction

Psoas abscess is regarded as a rare disease in the medical literature. In 1992, the worldwide reported occurrence of psoas abscess was 12 cases per year [1]. This was a significant increase from the calculated occurrence of 3.9 cases per year before 1985. Nowadays, the truly incidence is unknown but it has probably increased due to improvements in diagnostic techniques [2,3].

The causes of psoas abscess have also changed in the last decades. At the beginning of the 20th century, psoas abscess was mainly caused by tuberculosis (TB) of the spine (Pott's disease). With the decline of Mycobacterium tuberculosis (MT) as a major pathogen in developed countries, psoas abscess was mostly seen secondary to diseases of the digestive tract [2,4]. In recent years, a primary psoas abscess due to haematogenous spread from an occult source is more common, especially in immunocompromised and older patients [2,4]. In addition, TB is increasing again in some risk groups.

The psoas abscess may be clinically difficult to diagnose because of its rarity, insidious onset of the disease and nonspecific clinical presentation, which can cause diagnostic delays, resulting in high morbidity. Therefore, early diagnosis and appropriate management remain a challenge for clinicians.

We report a case of a primary psoas abscess due to MT infection in an immunocompetent female that presented with left lower abdominal pain and a soft tissue mass over the left iliac fossa (LIF) and inguinal regions.

## Case Presentation

A 64-year-old Portuguese white female was referred to the emergency department with progressive left lower quadrant abdominal pain radiating to the ipsilateral hip and thigh, over the previous 2 months. Palpable soft tissue mass over the LIF and inguinal areas was noted 1 week before admission. Her medical history was negative, except for high blood pressure. There was no history of trauma, cutaneous or other infection. The patient denied fever, back pain, gastrointestinal or genitourinary symptoms, weight loss or weakness.

On admission, her general condition was good and she was not febrile. The chest was clear to auscultation and no adenopathy was found. Examination of the LIF and inguinal regions showed a fluctuant, warm, mildly tender mass, measuring 10 × 5 cm. Flexion and external rotation of the ipsilateral hip was painful. The neurological examination was normal.

Blood test showed an increased C-reactive protein (1.48 mg/dl), without any others abnormalities. Urine analysis was normal.

**Figure 1:**
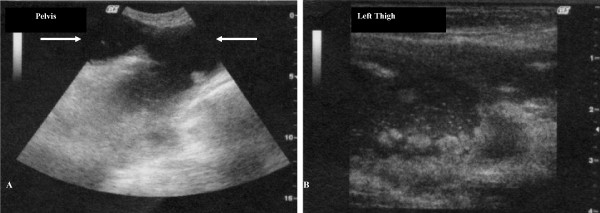
**Transverse abdominal, pelvic and left thigh ultrasound**. Hypoechoic collection with many internal echoes beginning on the left iliac fossa, involving the psoas muscle (arrows) **(A) **and extending inferiorly through the inguinal region until the root of the tight, with discrete infiltration of the subcutaneous tissues **(B)**.

Abdominal, pelvic and tight ultrasound (US) revealed the presence of a hypoechoic collection with many internal echoes (Figure. 1) and the computed tomography (CT) established the diagnosis of a loculated fluid-density mass, measuring 20 cm of length, beginning on the LIF, involving the psoas muscle and extending inferiorly through the inguinal region until the root of the tight, with discrete infiltration of the subcutaneous tissues.

The patient initiated broad spectrum intravenous antibiotherapy and percutaneous drainage (PCD) guided by US was performed, with aspiration of grossly purulent fluid. A catheter was placed for 6 days, obtaining 110 ml of pus. Microscopical examination of the fluid showed many polymorphonuclear cells without acid-fast bacilli and cultures for bacteria or fungi were negative. The microbiological study of blood, sputum and urine was also negative. There was no clinical evidence of other organs' involvement, namely the lung, the spine, the hip or the gastrointestinal or genitourinary tracts, which was also confirmed by radiological and endoscopic studies. Serologic HIV testing was negative.

A clinical and radiological improvement was seen, with the control CT, showing a residual collection confined to the subcutaneous region.

In the meanwhile, abscess culture was positive for *Mycobacterium tuberculosis complex *and the patient was oriented to our unit and started directly observed therapy with 4 weight adjusted drugs, (daily isoniazid 300 mg, rifampicin 600 mg, pyrazinamide 1500 mg and ethambutol 1200 mg). The specie was sensitive to all first line drugs in use. Two months later, the antituberculous treatment was reduced to 2 drugs, isoniazid and rifampicin. Almost after the 6 months of treatment, the patient is well and no signs of relapse have been detected.

## Discussion

The iliopsoas compartment is an extraperitoneal space which contains the iliopsoas and iliacus muscles. The psoas major muscle arises from the transverse processes and bodies of the 12^th ^thoracic and all lumbar vertebrae. Superiorly it passes beneath the arcuate ligament of the diaphragm, proceeds downward across the brim of the lesser pelvis, passes beneath the inguinal ligament and in front of the capsule of the hip joint and ends in a tendon that receives nearly the whole of the fibres of the iliacus muscle and is inserted into the lesser trochanter of the femur. Thus, the space defined by the psoas fascia is a direct communication from the mediastinum to the thigh. It is innervated by the branches of L2, 3, and 4, and it is the primary flexor of the hip joint.

The psoas abscess may be classified as primary or secondary, depending on the presence or absence of underlying disease. Since this muscle lies in close proximity to organs such as the sigmoid colon, appendix, jejunum, ureters, abdominal aorta, kidneys, pancreas, spine, and iliac lymph nodes, any underlying disease in these organs can spread secondarily to the iliopsoas muscle [4]. In a review of 367 cases, Ricci et al noted that the most common cause of secondary psoas abscess was Crohn's disease (60%) [5].

The etiology of primary psoas abscess is unclear, but lymphatic and hematogenous spread of an infectious process from an occult source in the body, often associated with immunosuppressant conditions like diabetes mellitus, renal failure, intravenous drug abuse, human immunodeficiency virus infection, malignancies and other chronic illness or trauma, has been implicated [4].

The review of Ricci et al noted differences in the aetiology of this entity. In secondary psoas abscess, cultures are often mixed with *Escherichia coli *and *Bacteroides *spp predominating [5]. On the other hand, *Staphylococcus aureus *is found in 88% of the cases of primary psoas abscess followed by streptococci (5%) and *Escherichia coli *(3%) [5]. In the literature, there are other reported causes of primary psoas abscess like brucellosis, trichinosis, typhilitis, pneumococcus or MT [6].

Iliopsoas abscess was first described by Mynter in 1881, who referred to this as psoitis; since then, iliopsoas abscess was characteristically a well recognised complication of TB of the spine [7]. Less frequently, tuberculous psoas abscess can be secondary to direct extension from other adjacent structures [8], or even haematogenous seeding from a distant site. However, the occurrence of tuberculous psoas abscess as the presenting manifestation of TB, without evidence of active infection elsewhere, has been seldom reported [6,9-12].

In the majority of these cases, as in our own case, those patients presented subacute or chronic symptoms and good general status. Although fever, abdominal or back pain and limitation of hip joint movements are the classical triad of psoas abscess, it can be presented only in 35% of all patients [3]. Clinical suspicion, radiological study with US and CT, the last considered the gold standard, along with microbiological culture of the pus, are crucial to the diagnosis.

Besides that, ruling out another source of infection in the lung, spine, hip, genitourinary or gastrointestinal tracts should be also kept in mind. Since no tuberculous disease was found in other organs, this case was considered as a primary psoas abscess. The respiratory tract might have been the route of entry of bacilli and in an adult the reactivation of a quiescent tuberculous focus from an occult source may result in haematogenous spread of bacteria, even in the absence of a predisposing condition.

Treatment of tuberculous psoas abscess involved the use of appropriate antituberculous drugs along with drainage. PCD has been shown to be the first line treatment of abdominal abscess, including tuberculous, which renders surgery unnecessary in many cases [13]. Our patient underwent US guided PCD and is responding well to antituberculous therapy, which is in keeping with most of the patients reviewed in literature, which reports that primary psoas abscess seems to have a better prognosis than those secondary to other disease [5].

## Abbreviations

CT: Computed tomography; LIF: Left iliac fossa; MT: Mycobacterium tuberculosis; PCD: Percutaneous drainage; TB: Tuberculosis; US: Ultrasound.

## Consent

Written informed consent was obtained from the patient for publication of this case report and accompanying images. A copy of the written consent is available for review by the Editor-in-Chief of this journal.

## Competing interests

The authors declare that they have no competing interests.

## Authors' contributions

APV, JG, JE, AC, RD analyzed and interpreted the patient data. They all gave a major contributor in writing the manuscript. All authors read and approved the final manuscript.
